# Indents

**Published:** 1901-03-15

**Authors:** 


### INDENTS.

☞ **Brief Articles of Technical or Scientific Value will receive notice and comment. Contributions invited.**

An Upper Fourth Molar.—Dr. Cryer called the attention of the New York Institute of Stomatology to the fact that he had noted in some cases a fourth molar developed. It was always in the upper jaw, he has never seen a lower fourth molar. He also ventured the assertion that there had been no deterioration of the human teeth during the past two thousand years.—New York letter in February *Dental Review*.

Kowarsko's Cement.—Dr. Hinkins, of Chicago, uses this cement to retain ligatures in place as splints for loose teeth. The teeth are ligatured together by tying them with several knots in each interproximal space until all are securely bound together and the spaces between the teeth are practically filled with the knots. Then the cement of a creamy consistence is applied in layers successively as it hardens. It should be applied much thicker than seems necessary as it shrinks greatly. It is made by dissolving *celluloid* in *acetone*.—*Dental Review*.

To Make Up for Use Cuttings of "Ideal Dental Base Plate."—Put the scrap into a small jar or pan, place this into a larger pan, which fill with sufficient water to cover the pan containing the base plate. Use for a lid an ordinary glazed tile or glass slab; boil until the base plate is quite soft. Place the base plate on the tile, which is now very hot; roll with a porcelain roller to desired thickness and cut into squares. The base plate is then not only as good as new, but even better, as it is less brittle. I trust the above may prove useful to your correspondent.—John Cromar, *The Dentist.*

Platinum Inlay Matrices.—Dr. Head, addressing the New York Odontological, states that he still finds platinum, not thicker than one one-thousandth of an inch, makes the best inlay matrix. He suggests time and care in adapting the platinum to the tooth cavity. After compressing the platinum into the cavity without undue force use a burnisher, starting at the edge of the cavity gradually burnish toward the center; don't burnish from the center outward, as you are liable to tear or wrinkle the platinum or the margins may project above the cavity, necessitating grinding to secure a fit.—New York letter in February *Dental Review.*

Kowarsko's Paste.—The paste is composed of acetone and celluloid, and may have a wide range ot usefulness. Use it on the sensitive neck of a tooth to protect it temporarily. If the tooth is dried with alcohol the liquid can be smeared over the neck ot the tooth or the crown, and it will dry in about fifteen minutes. It sticks to a tooth very firmly. It is made as follows:

| | |
|---|---|
| Acetone - - - - | gr. 500. |
| Celluloid - - - - - | gr. 155. |

This makes a stiff paste; it must be well mixed. It will harden sufficiently in about one hour and a half.—Dr. Hinkins, in *Dental Review.* [We should think it would make an inflammable composition.—Ed.]

TO REMOVE AMALGAM FILLINGS.—Dr. A. H. Brockway, of New York, says amalgam fillings may be more easily removed after applying a heated copper point to the filling for a few moments just previous to drilling it out.—New York letter in *Dental Review*. We have known of the application of mercury to these fillings for this same purpose, but the time necessary to make the application renders it impracticable, and we suspect the heated copper point will have no greater practical value. The best way to remove them is to use a well-sharpened Ambler drill in the engine, which will quickly perforate the hardest amalgam, and provide for easy use of a rose or fissure bur to completely remove the balance.

TIN AND GOLD; COLORATION.—Fillings of mixed tin and gold—one of tin to six of gold—will retain permanently the color of Roman gold, a pale greenish tint, laying a sheet of tin on three of gold and covering with three more of gold; Abbey's non-cohesive No. 4 and White's tin-foil No. 4. Cut in four or five strips, and then in suitable lengths, rolling between the fingers to form cylinders and avoiding exposure of the tin. In finishing the filling, burnishers must not be used or the color of the tin will be brought out. Simply polish with an instrument of copper or wood, with pumice powder. This must be done immediately, or the filling will become permanently dark.—A. Hugenschmidt, *Revue de Stomatologie*.

PAPAIN PASTE.—Dr. Harlan, in the February *Dental Review*, states that he does not believe the paste will decompose readily, as he has had a specimen in his possession for two months which still retains its virtue. The solvent may evaporate and leave the paste hard and dry. To use, remove all of the pulp practicable by instrumentation, then pack the paste into the canals and cover with paper fiber lint and seal the cavity with oxyphosphate cement, and leave in the tooth from two to three weeks. To remove the paste adjust the rubber dam and proceed with careful aseptic precautions to remove the filling and paste by instruments and solvent irrigants. If there are remnants of the pulp adhering to the canal wall remove with pliers or barbed broaches.

To Prevent Porcelain from Becoming Porous.—Dr. Head says: "There are very few porcelains that will not become porous if overheated. That is one of the most essential things, not to overheat the porcelain. It is not so difficult to fuse it as it should be fused, and a very little practice will make that a minor trouble. I have a piece of unbleached, unsized muslin, that I always keep to get my standard of moisture for my mixes. I take my colors as I think they ought to be mixed—as a painter would. After making that mass quite wet, I take three or four thicknesses of the unbleached muslin as I would a piece of blotting paper, and take out all the moisture that will come out. When I have done that I build on my contour, tap on my pliers, and the moisture flows to the surface; then I turn it upside down upon the muslin and allow all the excess moisture to be taken out again, and then I put it into the oven and allow it to become chalk white, which shows the moisture is all gone, and then I proceed to the fusing."—*Cosmos*.

